# Polycystic Liver With Cardiac Compression Leading to Atrial Fibrillation: Case Report and Review of the Literature

**DOI:** 10.7759/cureus.7976

**Published:** 2020-05-05

**Authors:** Ahmed Elfiky, Cheikh Talal El Imad, Boutros Karam, Stephen M Mulrooney

**Affiliations:** 1 Internal Medicine, Staten Island University Hospital - Northwell Health, Staten Island, USA; 2 Gastroenterology, Staten Island University Hospital - Northwell Health, Staten Island, USA; 3 Cardiology, Staten Island University Hospital - Northwell Health, Staten Island, USA

**Keywords:** polycystic liver, atrial fibrillation

## Abstract

Polycystic liver disease (PCLD) is a rare condition that most often occurs in patients with polycystic kidney disease and less commonly as an isolated liver disease. Complications include cyst rupture, infection, hemorrhage, and compression of surrounding organs by large cysts. We present the case of a patient with a history of PCLD who presented to our hospital with palpitations and was found to have atrial fibrillation. Imaging and echocardiograph revealed a dominant large cyst compressing the right atrium. Other etiologies including thyroid disease, ischemic heart disease, and electrolytes abnormalities were excluded. The patient refused surgical intervention and was conservatively treated with rate control and anticoagulation. To the best of our knowledge, this is the first case of new-onset atrial fibrillation secondary to right atrial compression by a liver cyst. Compression of cardiac chambers resulting in new-onset arrhythmia should be considered when evaluating patients with PCLD.

## Introduction

Polycystic liver disease (PCLD) is commonly associated with autosomal dominant polycystic kidney disease (ADPKD) [1]. Most liver cysts are asymptomatic and require no treatment [2]. Complications include infection, rupture, hemorrhage, and compression of surrounding organs [3]. We here present the first case of polycystic liver compressing the right atrium leading to new-onset atrial fibrillation.

## Case presentation

**Figure 1:**
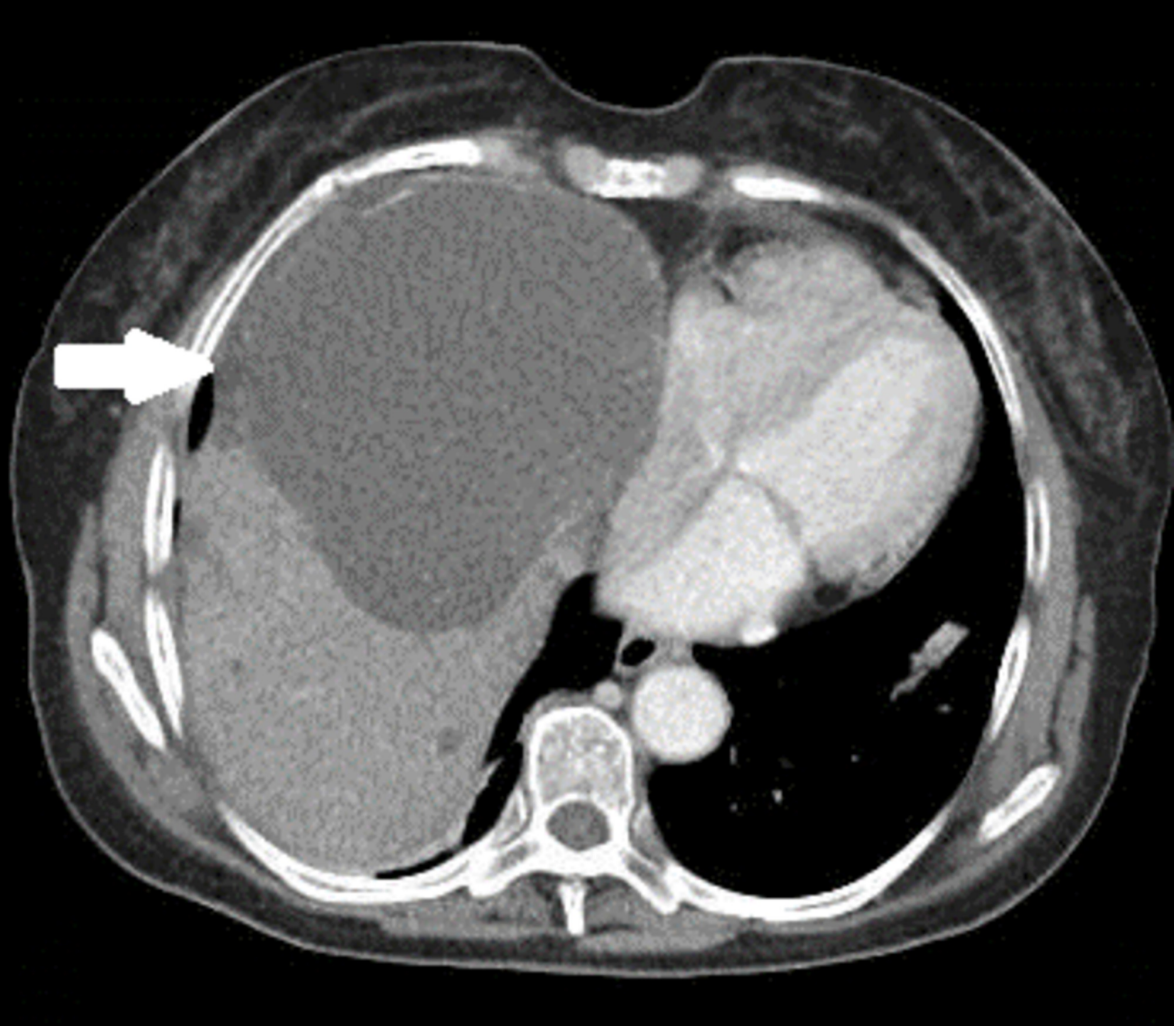
Transverse section of computed tomography (CT) scan of the abdomen and pelvis, with the arrow indicating a large liver cyst compressing the right atrium of the heart

**Figure 2:**
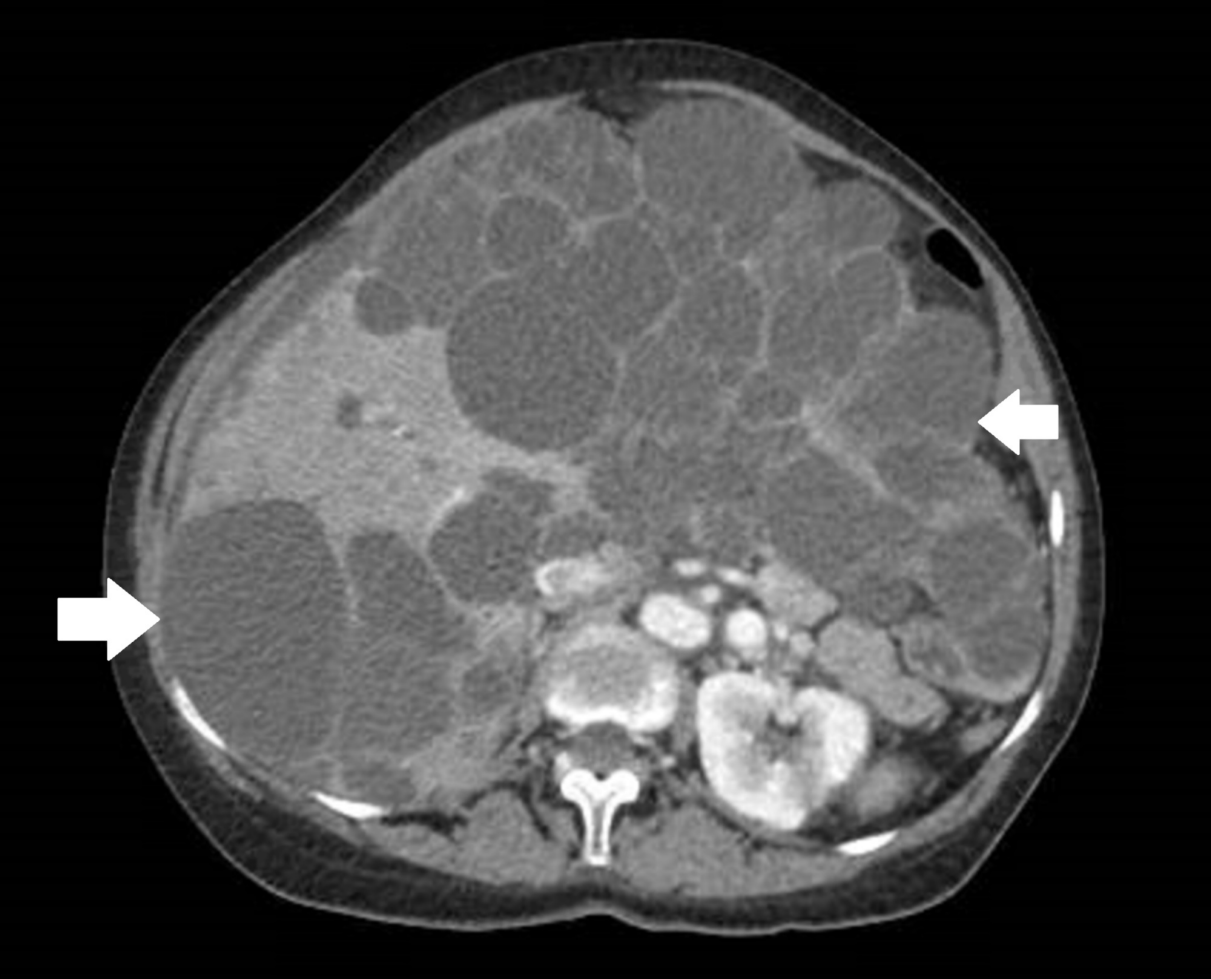
Transverse section of computed tomography (CT) scan of the abdomen and pelvis, with the arrows indicating innumerable cysts

A 75-year-old female patient presented to our emergency department for palpitations. Her review of system was positive for intermittent right-sided chest pain and palpitation. Her medical history revealed hypertension, PCLD, and macular degeneration. Her family history is significant for PCLD. Vital signs upon presentation were notable for heart rate of 124 beats per minute, blood pressure of 143/81 mm Hg, temperature of 97.1°F, and respiratory rate of 18 breaths per minute. Physical examination was unremarkable except for marked hepatomegaly on abdominal palpation and rapid irregular pulse. An electrocardiogram performed at presentation showed a new-onset atrial fibrillation with rapid ventricular response. The patient received 1 L of lactated ringer and was started on a beta-blocker, which resulted in heart rate control. Computed tomography (CT) scan of the abdomen and pelvis performed to evaluate for hepatomegaly revealed a markedly enlarged liver containing innumerable cysts, largest measuring 12.62 cm by 10.56 cm and compressing the right atrium (Figures 1, 2).

**Figure 3:**
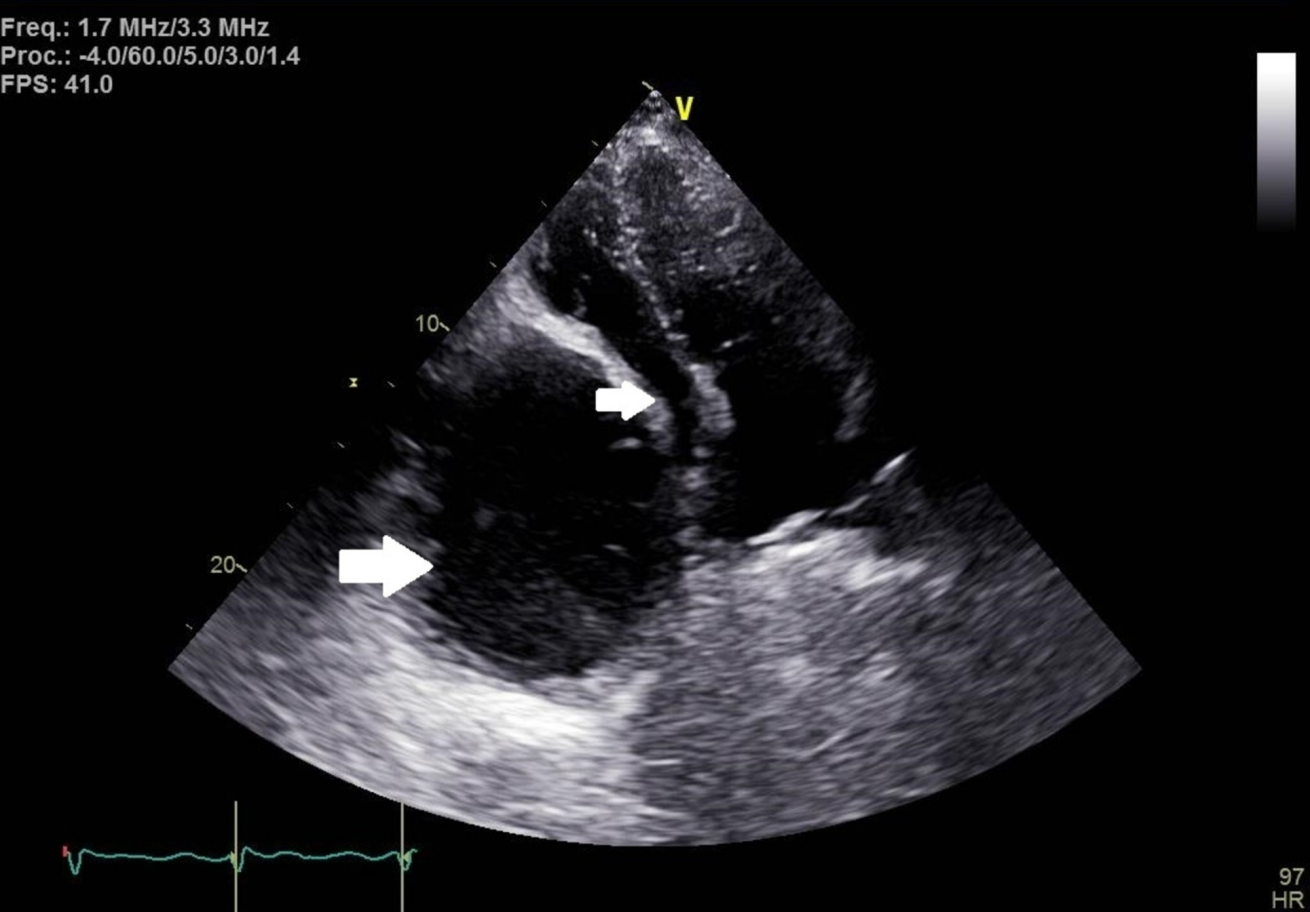
Transthoracic echocardiograph with apical four-chamber view, with the upper arrow indicating the right atrium and the lower arrow indicating liver cyst compressing the right atrium

**Figure 4:**
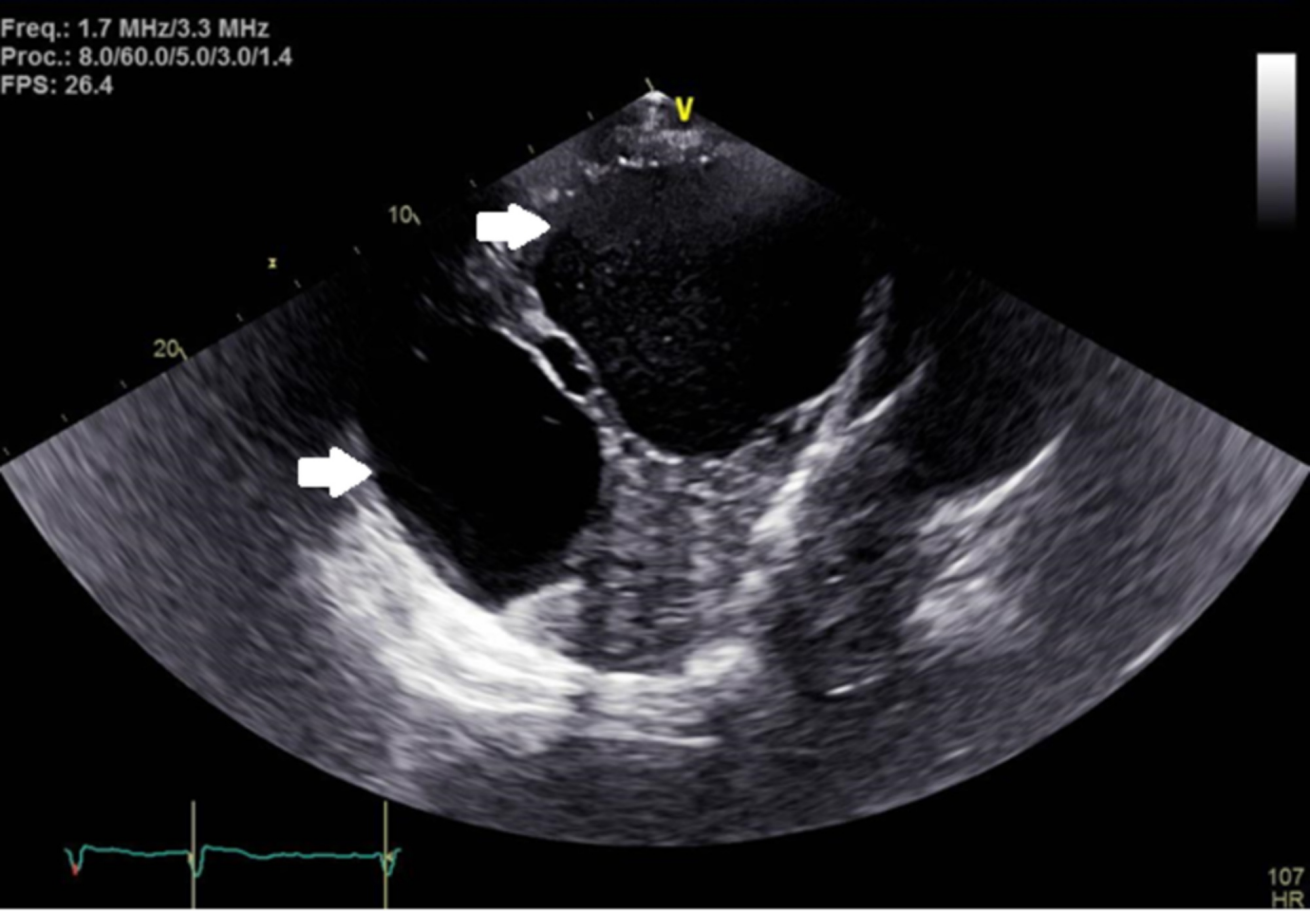
Transthoracic echocardiograph with a subxiphoid view, with arrows indicating liver cysts

Transthoracic echocardiograph performed showed a large cystic mass in the right upper quadrant compressing the right atrium (Figures 3, 4).

Apart from an isolated mild elevation of serum alkaline phosphatase of 229 U/L, the hepatic function panel was unremarkable. Renal functions test revealed serum blood urea nitrogen of 17 mg/dL and creatinine of 0.7 mg/dL. Thyroid function test was unremarkable. Serum electrolytes including potassium and magnesium were within the normal range. A nuclear stress test was performed for the evaluation of intermittent right-sided chest pain revealed no evidence of ischemic heart disease. The patient was started on anticoagulation and beta-blocker as rate control agents. The patient declined surgical intervention. She was subsequently discharged on hospital day 3. The patient has been medically stable on conservative treatment.

## Discussion

PCLD is most commonly associated with polycystic kidney disease. An uncommon form is isolated PCLD, typically autosomal dominant. Around 70 to 90 % of patients with ADPKD have PCLD, but PCLD without adult polycystic kidney disease has a prevalence of less than 10% [1]. In our case, the presence of multiple liver and kidney cysts on imaging is suggestive of a diagnosis of ADPKD with associated PCLD. Polycystic liver cysts are fluid-filled biliary epithelial cysts that usually result from malformation in the intrahepatic bile duct system during embryonic liver development. Polycystic kidney disease 1 (PKD1) and polycystic kidney disease 2 (PKD2) genes are associated with polycystic kidney disease form, whereas protein kinase C substrate 80k-H (PRKCSH) and translocation protein SE (SEC63) genes are associated with dominant PCLD [2]. Cardiovascular associations with polycystic liver include mitral valve prolapse, abdominal aortic aneurysm, and aortic root dilatation, which have a higher prevalence in ADPKD up to 25-41.2% compared with 0-10.5% in PCLD [1]. Macutkiewicz et al. reported several complications associated with PCLD, including compression of surrounding structures such as inferior vena cava obstruction, common bile duct obstruction, and portal vein occlusion [3]. Compression of the heart by hepatic cyst has been reported in the medical literature, most commonly presenting as right-sided heart failure [4-7]. Ker reported a case of a simple liver cyst with occasional right atrium compression on echocardiograph, resulting in atrial premature beats [8]. Sanchez-Recalde et al. reported a case of hepatic hydatid cyst compressing the right atrium on echocardiograph in a patient presenting with common atrial flutter with variable atrioventricular conduction. Surgical interventions with cyst resection achieved conversion to sinus rhythm in the immediate post-operative period and at six months follow-up [9]. In our case, other etiologies for atrial fibrillation such as thyroid disease, ischemic heart disease, and electrolytes abnormalities were excluded. Treatment of PCLD is warranted in symptomatic patients. Treatment options include somatostatin analogues, which have been proven beneficial as pharmacotherapy especially in young females [10]. Withholding estrogen therapy is indicated in females as estrogen therapy has been associated with increasing liver cyst size [2]. Surgical treatment includes aspiration sclerotherapy for cysts more than 5 cm, laparoscopic keyhole fenestration for multiple large hepatic cysts, and segmental hepatic resection. Liver transplantation is indicated in untreatable complications, progressive hepatomegaly, and end-stage liver disease.

## Conclusions

Compression of the surrounding organs by liver cysts is a reported complication of PCLD. Compression of cardiac chambers resulting in new-onset arrhythmia should be considered when evaluating patients with PCLD. To the best of our knowledge, this is the first report of a case of a new-onset atrial fibrillation secondary to right atrial compression by a liver cyst.
